# High efficiency and stability of ink-jet printed quantum dot light emitting diodes

**DOI:** 10.1038/s41467-020-15481-9

**Published:** 2020-04-02

**Authors:** Chaoyu Xiang, Longjia Wu, Zizhe Lu, Menglin Li, Yanwei Wen, Yixing Yang, Wenyong Liu, Ting Zhang, Weiran Cao, Sai-Wing Tsang, Bin Shan, Xiaolin Yan, Lei Qian

**Affiliations:** 1 TCL Research, No. 1001 Zhongshan Park Road, Nanshan District, Shenzhen, 518067 People’s Republic of China; 2 Division of Functional Materials and Nanodevices, Ningbo Institute of Materials Technology and Engineering, Chinese Academy of Science, 1219 West Zhongguan Road, Ningbo, 315201 Zhejiang People’s Republic of China; 3 Center of Super-Diamond and Advanced Films (COSDAF), City University of Hong Kong, Tat Chee Avenue, Kowloon, Hong Kong SAR People’s Republic of China; 4 Department of Materials Science and Engineering, City University of Hong Kong, Tat Chee Avenue, Kowloon, Hong Kong SAR People’s Republic of China; 5 State Key Laboratory of Materials Processing and Die & Mould Technology, School of Materials Science and Engineering, Huazhong University of Science and Technology, 1037 Luoyu Road, Wuhan, 430074 Hubei People’s Republic of China

**Keywords:** Quantum dots, Organic LEDs

## Abstract

The low efficiency and fast degradation of devices from ink-jet printing process hinders the application of quantum dot light emitting diodes on next generation displays. Passivating the trap states caused by both anion and cation under-coordinated sites on the quantum dot surface with proper ligands for ink-jet printing processing reminds a problem. Here we show, by adapting the idea of dual ionic passivation of quantum dots, ink-jet printed quantum dot light emitting diodes with an external quantum efficiency over 16% and half lifetime of more than 1,721,000 hours were reported for the first time. The liquid phase exchange of ligands fulfills the requirements of ink-jet printing processing for possible mass production. And the performance from ink-jet printed quantum dot light emitting diodes truly opens the gate of quantum dot light emitting diode application for industry.

## Abstract (web summary)

Designing efficient and scalable quantum dot LEDs meeting industrial requirements remains a challenge. Here, the authors, by leveraging the liquid phase exchange of d-MX_2_ ligands, present printed quantum dot LEDs with external quantum efficiency over 16% and half lifetime of more than 1,721,000 hours.

## Introduction

Colloidal quantum dots (CQDs), due to their bandgap tunability via quantum size effect, have been demonstrated as excellent semiconducting materials for optoelectronic devices and applications such as quantum dot light emitting diodes (QLEDs)^1–5^, photodetectors^6–8^, and photovoltaics^9–12^. Together with solution processing capability, CQDs serve as preferable candidates for inkjet printing (IJP) manufacturing for the next generation QLED display application^13–15^. IJP is a drop-on-demand technology, which promises a mask free patterning and low material consumption processing. In display industry, IJP has demonstrated that it could solve the issues caused by fine metal mask of large area fabrication and potentially reduce the manufacturing cost, thus has drawn a lot of interests and investments from both academy and industry^16–18^.

However, the low efficiency and fast degradation of CQDs emission during QLED operation fail to meet the display requirements. The increase of non-radiative recombination of CQDs is strongly depending on the trap state energy and density^19^. Extensive engineering of core/shell structures has been carried out in previous reports including gradient intermedium shell and smaller energy gap shell martials. With these methods, not only the external quantum efficiency (EQE) over 20% has been achieved but also half lifetime of QLED device has reached over 2,000,000 h^1–3,19^.

Besides tailoring the energy band structures of core and shell of CQDs, ligands also play a magnificent role in trap-related problems^20–23^. Surface states are found to have more influence on the core emission. The trap states from the shell surface can quench the core emission. Even with a thicker shell, surface trap states are present within the bandgap due to the high surface to volume ratio of CQD. These mid-gap trap states, tending to recombine carriers and quench excitons, are considered as non-radiative recombination centers^9,11^. To better eliminate trap states, both cations and anions need to be passivated, especially the anions. Even though, several studies tried different types of ligands to eliminate trap states for better device efficiency^19,24,25^, there is no effort to passivate both ionic traps in QLEDs.

On the other hand, ligands play important roles in the compatibility of the inkjet printing solution process. Solid phase ligand exchange is hard to implement in large area mass production^9,19,25^. For core/shell CQDs, although halide salt can passivate the anion, it is difficult to achieve complete ligand exchange either during the shell synthesis or afterwards in liquid phase^26^. Alternatively, mixed passivation by two ligands was also proposed^11,12^, however, due to the relatively low ligand exchange coverage and the negative influence on optoelectrical property, it is challenging to obtain both sufficient passivation for dual traps and good emitting property of CQDs.

Based on the analysis, we proposed a type of MX_2_ ligand^27^ to passivate our zinc selenide (ZnSe) shell based CQDs, aiming higher device performances. It is worth noting that this type of MX_2_ (d-MX_2_) is capable of passivating both cations and anions. M cations are in favor of bonding with the under-coordinated selenium (Se) anion sites, while the X group tends to form bonds with the under-coordinated Zn sites on the CQD surface. More importantly, regarding device performance, the binding energy of the ligand link to the CQD surface should also be considered. The stability of the surface ligand can be measured by the binding energy. Stronger binding energy and more binding bonds promise better stability during device operation and longer lifetime. Moreover, the proposed d-MX_2_ ligands we chose are suitable for liquid phase ligand exchange. d-MX_2_ type ligand with an organic electron donor group settles the issues of passivation and devices fabrication processing. Thanks to alkene/alkane long chain organic group, Zn(OA)_2_ ligand shows better solubility, which favors liquid phase ligand exchange. Together with the benefits from the efficient trap states passivation and stronger binding, we demonstrated that IJP processed QLED could achieve EQE of 16.6% and operation lifetime (LT_95_@1000nits) of 1833 h.

## Results

### Simulation of the surface trap states

**Fig. 1:**
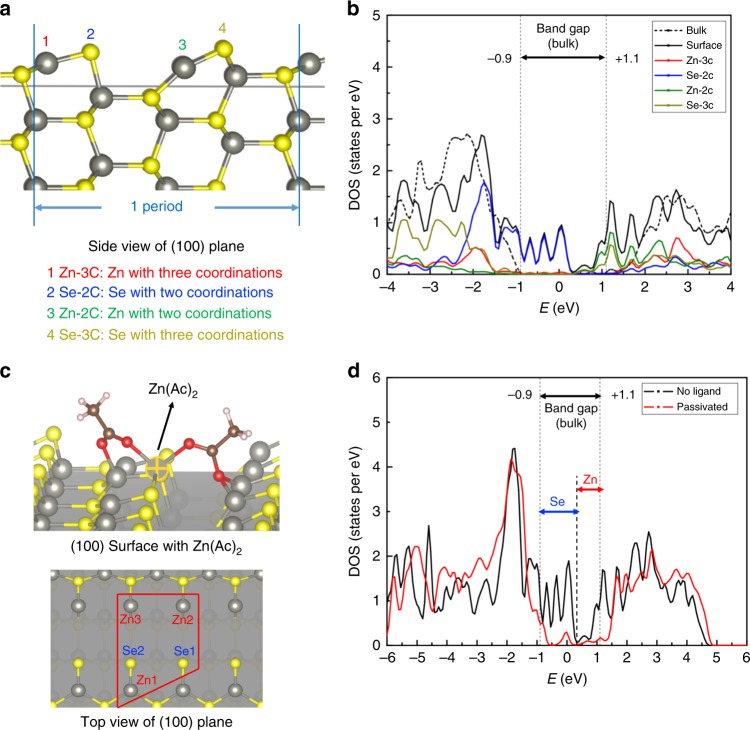
Simulation of QD surface. **a** The under-coordinated Zn and Se atoms on Wurtzite (100) ZnSe surface. **b** Density of states (DOS) of bulk ZnSe, and projected density of states (pDOS) of (100) ZnSe surface and under-coordinated Zn and Se atoms on (100) surface. **c** The bonding configuration of Zn(Ac)_2_ to ZnSe (100) surface. Zn1, Zn2, Zn3, Se1, and Se2 are the bonding sites of Zn(Ac)_2_ on ZnSe (100) surface. Top figure is the 3D illumination of the bonding configurations, and the bottom figure is the top view of the bonding sites of atoms on the (100) surface. **d** The density of states (DOS) before and after Zn(Ac)_2_ passivation, the Se and Zn labels indicate which atom are most responsible for the specific reduction of mid-gap states.

We simulated shell surface of CQDs using density function theory (DFT) to better understand the trap states. X-ray diffraction (XRD) confirmed that the crystal structure of our QDs is wurtzite (Supplementary Fig. 1), therefore the (100) wurtzite ZnSe surface model is chosen to study the trap states induced by under-coordinated anion and cation sties on the surface. Different types of under-coordinated cations and anions are shown in Fig. 1a, b show the density of states (DOS) of bulk material and projected density of states (pDOS) of ZnSe (100) surface. Here, the DOS (dash black line) shows a clean bandgap of the bulk ZnSe. In case of the (100) ZnSe surface where atoms are under-coordinated, trap states are introduced into the bandgap with the present of dangling bonds (solid black line). The pDOS (colored solid lines) is used to analyze which under-coordinated surface atom contributes to the mid-gap trap states. As shown in Fig. 1b, the states near the valence band are mainly caused by the two coordinated Se-2C atoms. The two coordinated Zn-2C atoms and the three coordinated Se-3C atoms mainly contribute to the states near conduction band. As a result, the two coordinated surface Se-2C atoms lead to hole traps. While both the two coordinated Zn-2C atoms and the three coordinated Se-3C atoms lead to electron traps. Moreover, the two coordinated surface Se-2C atoms introduce more states into the bandgap.

Zinc Oleate (Zn(OA)_2_) is chosen as the typical MX_2_ ligand of our proposal. However, given the computing efficiency and the little difference between zinc oleate and zinc acetate (Zn(Ac)_2_) in DFT simulation of ligand passivation, we chose the simpler structural Zn(Ac)_2_ in our previous simulated (100) ZnSe surface to illustrate the effect of d-MX_2_. The top picture of Fig. 1c shows the lowest energy configuration of how Zn(Ac)_2_ bonds to ZnSe surface. In this configuration, Zn(Ac)_2_ molecule bonds to two Se atoms and three Zn atoms. These atoms are the under-coordinated ones causing the traps in the mid-gap. Their positions are shown in the bottom picture of Fig. 1c. As the results, the trap states caused by Se and Zn atoms are both reduced after target passivation, as shown in Fig. 1d. Compared with the un-passivated surface, 84% of the mid-gap states are eliminated. As comparison, the simulation results of commonly used CH_3_COOH, and CH_3_SH molecules are presented in Supplementary Fig. 2. Significantly low mid-gap states removal was observed because that they can only passivate one ion with no noticeable reduction of the trap states caused by the other ion. The binding energy of these molecules on (100) ZnSe surface was also calculated. Table 1 shows the binding energy of different types of ligands. The Zn(Ac)_2_ has the highest binding energy of −4.1 eV, which shows 60.2% and 43.4% increases comparing with CH_3_COOH and CH_3_SH molecules, respectively.

**Table 1:** Binding energy of different ligands on (100) ZnSe surface.

Ligand	Zn (CH_3_COO)_2_	CH_3_COOH	CH_3_SH
Binding energy (eV)	−4.1	−2.56	−2.86

### Characterization of the ligands binding to the surface

**Fig. 2:**
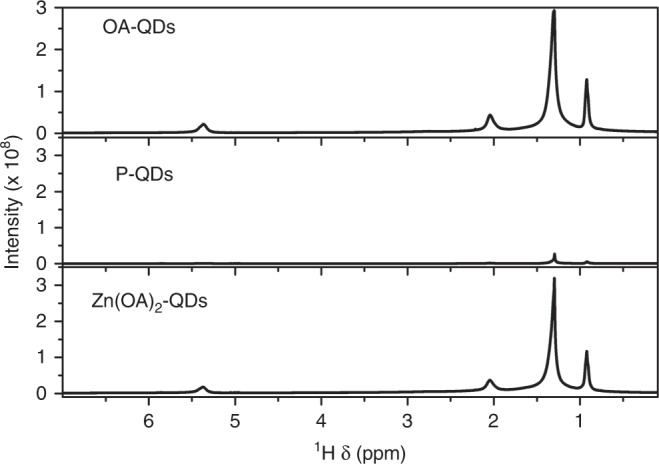
Nuclear Magenetic Resonance Measurements. Nuclear magnetic resonance (^1^HNMR) spectra of different ligands.

In this study, CdSe core and ZnSe shell QDs were synthesized according to the previous method^2^. After further treatment (see Materials in the “Methods” section for details), the purified QDs (referred as P-QDs) with negligible ligands coated on the surface were obtained. Then through solution ligand exchange procedure, cation passivated CQDs (oleic acid (OA)-coated QDs, referred as OA-QDs), anion passivated CQDs (zinc chloride-coated QDs, referred as ZnCl_2_-QDs), and our dual-ion passivated CQDs (zinc oleate coated QDs, referred as Zn(OA)_2_-QDs) were produced separately. Nuclear magnetic resonance (^1^HNMR) spectra confirmed the treatment of ligands in Fig. 2. ^1^HNMR spectra show the characteristic signals of OA- on the OA-QDs sample, where saturated chains show the methyl group at about 0.9 ppm and the rest of the methylene CH_2_ groups overlapping at about 1.3 and 2.0 ppm. OA has one double bound which is coupled to a single olefinic hydrogen with a signal at about 5.3 ppm. After purification, the characteristic signals almost disappeared, indicating the removal of OA- ligands. And these signals emerged back after the solution ligand exchange procedure using Zn(OA)_2_. High-resolution transmission electron microscopy (TEM) was used to track the QDs size and morphology changes before and after the ligand exchange. As shown in Supplementary Fig. 3, the ligand exchange has no noticeable influence on the size and morphology of the QDs. All the ligand coated QDs have the similar spherical shape and particle sizes around 12 nm.

**Fig. 3:**
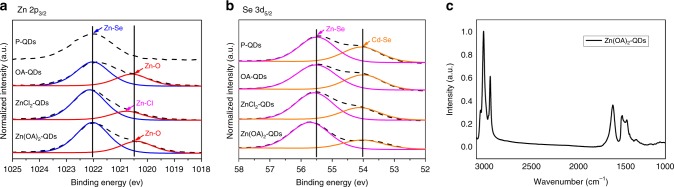
Characterization of ligand binding states on QD surface. **a** Zn 2*p*_3/2_ spectra and **b** Se 3*d*_5/2_ spectra from X-ray photo-electronic spectroscopy measurement of all kinds of ligand coated QDs. **c** Fourier transform infrared spectroscopy measurement of Zn(OA)_2_-QDs.

To confirm the effectiveness of the ligand exchange, X-ray photoelectron spectroscopy (XPS) was used to investigate the chemical component of the QDs with different ligands. The Zn 2*p*_3/2_ and Se 3*d*_5/2_ characteristic peaks of all ligand coated QDs are shown in Fig. 3a, b, respectively. According to the reference^28^, the Zn–Se bond in the Zn 2*p*_3/2_ spectra should have a peak at 1022.3 eV, corresponding well to the dominant peak of P-QDs in Fig. 3a. For the other three kinds of ligand coated QDs, their Zn 2*p*_3/2_ spectra can be well fitted by two oxidation states in the form of a Gaussian function. Except for the dominant peak at 1022.0 eV corresponding to the Zn–Se bond, the other peak at lower energy should be resulted from surface cation (Zn) passivation by ligands. Although the binding energy of the coordination bond could not be found from the reference, considering the binding strength of different types of the coordination bond and the possibility of the coordination bond formation in each type of ligands, the minor peak at 1020.6 eV for both OA-QDs and Zn(OA)_2_-QDs in the Zn 2*p*_3/2_ spectra should be corresponding to the Zn–O coordination bond, and the clear shift toward higher binding energy of this minor peak from ZnCl_2_-QDs indicates the presence of Zn–Cl coordination bond. As calculated from the integral area in the Zn 2*p*_3/2_ spectra, the concentration ratio of Zn–Se sites vs. Zn surface passivated sites is 1:0.34 for OA-QDs, 1:0.19 for ZnCl_2_-QDs, and 1:0.45 for Zn(OA)_2_-QDs, respectively. The low ratio of ZnCl_2_-QDs reveals the limited surface cation passivation by liquid phase exchanged ZnCl_2_ ligand. The high ratio of Zn(OA)_2_-QDs indicates the improved cation passivation by Zn(OA)_2_. On the other hand, the Se 3*d*_5/2_ spectra for all samples can be fitted with two components in Fig. 3b. Given the investigation depth of XPS technique, the major peak around 55.4 eV is contributed by the Zn–Se bond from the outer shell, and the minor peak around 54.0 eV could be attributed to the Cd–Se bond from the medium shell of the QDs^28^. After calculation, the concentration ratio of Cd–Se sites and Zn–Se sites is 1:1.52 for P-QDs, 1:1.56 for OA-QDs, 1:1.91 for ZnCl_2_-QDs, and 1:2.72 for Zn(OA)_2_-QDs, respectively. There is no significant difference between p-QDs and OA-QDs, because OA cannot passivate Se sites. ZnCl_2_-QDs show limited passivation due to unfavorable liquid phase exchange process. The largely increased Zn–Se sites of Zn(OA)_2_-QDs directly demonstrate the greater surface anion passivation by Zn(OA)_2_. The bidentate carboxylate binding environment of Zn(OA)_2_-QD surface is also observed by Fourier transform infrared spectrometer (FTIR) in Fig. 3c. The FTIR spectrum of Zn(OA)_2_-QDs has a broad alkane/alkene C–H stretches around 2740–3000 cm^−1^, and a bidentate carboxylate binding environment is observed with symmetric and asymmetric COO–QD stretches at 1558 and 1403 cm^−1^. Combining the XPS results for both Zn 2*p*_3/2_ and Se 3*d*_5/2_ spectra, Zn(OA)_2_ demonstrated its capability of dual-ion surface passivation.

**Fig. 4:**
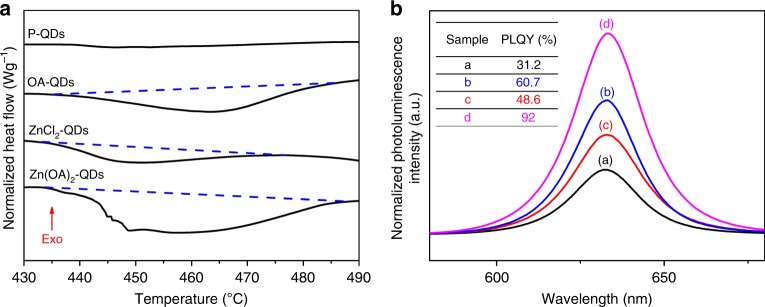
The significance of surface ligands passivation on quantum dots. **a** Differential scanning calorimetry measurement for all kinds of ligand coated quantum dots. Upper direction, as marked by the arrow, represents exothermal heat flow. **b** Photoluminescence intensity of all kinds of ligand coated colloidal quantum dots. **a** P-QDs, **b** OA-QDs, **c** ZnCl_2_-QDs, and **d** Zn(OA)_2_-QDs. Inserted table shows the photoluminescence quantum yield of all colloidal quantum dots in octane.

High-resolution differential scanning calorimetry has been carried out to test the binding energy of each type of ligand on the QDs. As shown in Fig. 4a, except for the negligible heat flow of P-QDs, a clear endothermic peak between 440 and 480 °C has been observed for all ligand coated QDs. Due to the absence of phase transition of QDs at this temperature range and the much lower evaporation temperature for each type of ligands, the endothermic peak characterized enthalpy changes during the surface ligand detachment under the inertial environment during the DSC measurement, which represents the binding energy of surface ligands to the QDs. After subtracting the baseline and integrating the peak, the endothermic peak areas for each sample are 56.92 J g^−1^ for OA-QDs, 23.19 J g^−1^ for ZnCl_2_-QDs, and 97.76 J g^−1^ for Zn(OA)_2_-QDs, respectively. The largest endothermic peak area of Zn(OA)_2_-QDs experimentally indicated the highest binding energy of Zn(OA)_2_ ligand attached to our QDs surface, corresponding well to our simulation results. Moreover, besides the better stability introduced by the highest binding energy of Zn(OA)_2_ ligand, the significantly reduced trap states resulted from the dual-ion passivation of Zn(OA)_2_ lead to a great improvement on the PLQY of our CQDs, evidenced by the photoluminescence measurement and PLQY results as shown in Fig. 4b.

### Comparison of IJP devices with different ligands

**Fig. 5:**
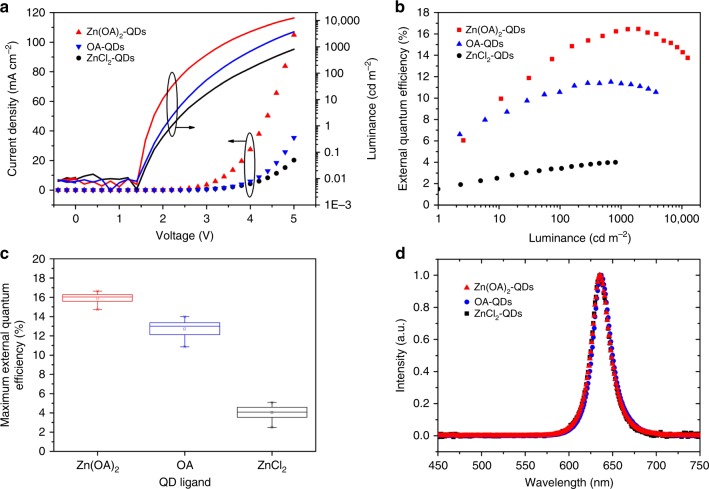
IJP QLED characterizations. **a** Current–voltage–luminance characterization of Zn(OA)_2_-QDs vs. OA-QDs vs. ZnCl_2_-QDs devices; **b** external quantum efficiency of Zn(OA)_2_-QDs vs. OA-QDs vs. ZnCl_2_-QDs devices; **c** statistic chart of maximum external quantum efficiency of devices; **d** electroluminescent spectra of Zn(OA)_2_-QDs vs. OA-QDs vs. ZnCl_2_-QDs devices.

**Fig. 6:**
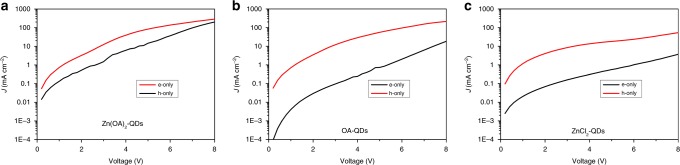
Single Carrier Devices. Current density vs. Voltage plots of Single carrier devices: **a** Zn(OA)_2_-QDs, **b** OA-QDs, and **c** ZnCl_2_-QDs.

Our dual-ion passivated CQDs were implemented into state-of-art QLED architectures through IJP process. QLEDs are bottom emission devices. On top of indium-tin oxide (ITO), we inkjet printed commercial materials Nissan HIL, TFB, CQDs, and ZnO nanoparticles, as hole injecting layer, hole transporting layer, emitting layer, and electron transporting layer, respectively. Finally, aluminum was evaporated on top as cathode. Supplementary Fig. 4 shows the cross-section imagine of a typical device by scanning electron microscope. The pixel array image of an operating Zn(OA)_2_-QDs device is presented in Supplementary Fig. 5. We also fabricated devices with ZnCl_2_-QDs and OA-QDs as comparisons. Through current–voltage–luminance measurement, QLEDs with different ligands were characterized. As shown in Fig. 5a, all devices’ turn-on voltages are around 2 V which indicates a direct band edge injection of carriers. Zn(OA)_2_-QDs device has the highest current density, while ZnCl_2_-QDs one shows the lowest current density. This can be explained with space charge limit current theory. Considering the energy level tuning from different ligands is insignificant, the current density will be determined by trap density. With better passivation of ions, Zn(OA)_2_-QDs shows the highest current density. We also fabricated single carrier devices to study the influence of different ligands. The results of them are shown in Fig. 6d. Previously, the better injection from ZnO were thought to be responsible for electrons as the dominate carriers in an QLED structure. But hole current is orders of lower^2^. However, the influence of ligand passivation is underrated. Good cation passivation from organic ligands also plays a part in high electron current. This is the situation of OA-QDs devices. Meanwhile, ZnCl_2_-QDs is supposed to be a hole trap passivation. Therefore, we observed a low electron current. However, due to the insufficient passivation by liquid exchange, insignificant hole current improvement was observed in ZnCl_2_-QDs hole only device. In case of Zn(OA)_2_-QDs, through our dual ion passivation, anion traps are effectively passivated, resulting a significant improvement of hole current. As the results, we have better balanced currents in Zn(OA)_2_-QDs devices. With higher PLQY and better charge balance, QLED with Zn(OA)_2_-QDs achieved the highest EQE of 16.6% (Fig. 5b). As far as we know, this is the first time that reported an IJP QLED device with EQE over 15%. The average maximum EQE of each QD device is shown in Fig. 5c. The ligands we chose here have weak effect on the energy level of CQDs, thus electroluminescent spectra are the same of all types of ligands (Fig. 5d).

**Fig. 7:**
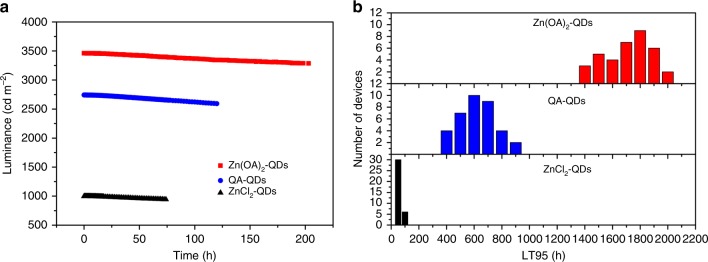
IJP devices lifetime. **a** Lifetime of OA-QDs, ZnCl_2_-QDs, and Zn(OA)_2_-QDs devices under 25 mA cm^−2^; **b** statistics of device performance. Histograms of extrapolated LT_95_ at an initial brightness of 1000 cd m^−2^ of OA-QDs, ZnCl_2_-QDs, and Zn(OA)_2_-QDs devices with 36 devices for each QD.

We also characterized the operation lifetime of different ligand devices under constant current density. The comparison of luminance vs. operating time for Zn(OA)_2_-QDs, OA-QDs, and ZnCl_2_-QDs devices is presented in Fig. 7a. All devices were driven at a constant current density of 25 mA cm^−2^ under temperature of 21–23 °C with glass cap encapsulations. Initial luminance of the Zn(OA)_2_-QDs device, OA-QDs device, and ZnCl_2_-QDs device is 3462, 2741, and 995 cd m^−2^, respectively, which are consistent with EQE values. The luminance of the devices drop to 95% of its initial value (LT_95_) is compared. The Zn(OA)_2_-QDs device has the longest LT_95_, which is over 196 h, while it takes <70 h for the ZnCl_2_-QDs device reaching its LT95. And LT_95_ of OA-QDs device is 113 h. The extrapolated LT_95_ at an initial brightness of 1000 cd m^−2^ of Zn(OA)_2_-QDs devices is 1833 h, with acceleration factor of *n* = 1.80 by fitting LT_95_ values at various luminance (Supplementary Fig. 6a). To better compare the lifetime, we also converted the lifetime results of OA-QDs and ZnCl_2_-QDs devices to the initial brightness of 1000 cd m^−2^. Reproducibility of 36 devices was shown in the histograms of the lifetime (Fig. 7b) of each Zn(OA)_2_-QDs, OA-QDs, and ZnCl_2_-QDs device. The lifetime of Zn(OA)_2_-QDs is from 1400 to 2000 h which is two times larger than OA-QDs. While the lifetime of most ZnCl_2_-QDs device is <100 h. The average lifetime of OA-QDs and ZnCl_2_-QDs devices is 671 and 79 h, respectively. In case of Zn(OA)_2_-QDs, we obtained an average LT_95_ lifetime at 1000 cd m^−2^ of 1769 h. For a direct comparison with previously reported values of QLED lifetime, a half (LT_50_) lifetime with initial luminance of 100 cd m^−2^ can be estimated to be 1,721,000 h (Supplementary Fig. 6b), which is in the same order of magnitude of reported spin-coating devices^2,29^.

### Device degradation mechanism investigation

**Fig. 8:**
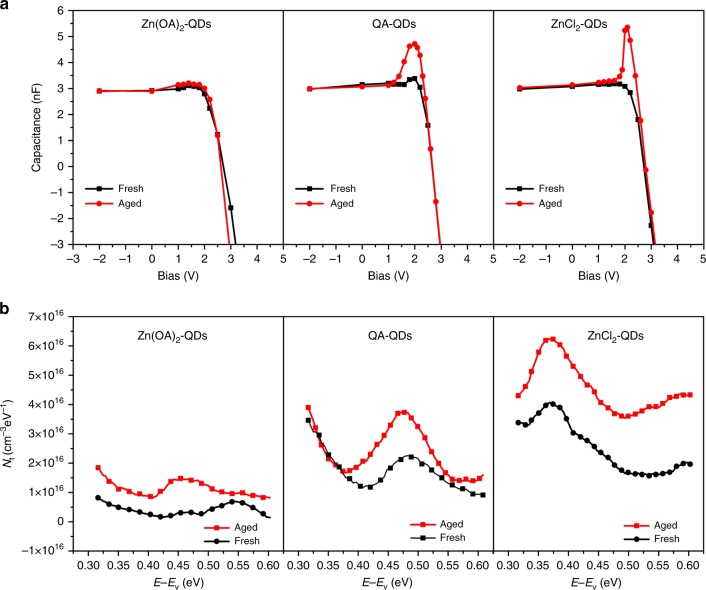
Characterizations of device degradation mechanism. **a** Capacitance–voltage (CV) measurements and **b** density of state (DOS) calculated from thermal admittance spectroscopy (TAS) before and after aging.

We further investigated the degradation mechanism behind the varieties of lifetime between those devices with different surface passivation strategies using the capacitance–voltage (CV) measurements and thermal admittance spectroscopy (TAS). First, CV measurements were conducted on those devices before and after aging. As shown in Fig. 8a, there is negligible change of capacitance, <0.1 nF, for Zn(OA)_2_-QDs devices after 20 h operation. On the other hand, significant increase of capacitance was observed in ZnCl_2_-QDs and OA-QDs devices after aging. The capacitance of OA-QDs device raised from 3.34 to 4.94 nF and ZnCl_2_-QDs device from 3.03 to 5.27 nF, suggesting that more traps were formed during the operation which further deteriorate the device performance. As the three devices were using the same interlayer materials, we hypothesized that such increase of traps was due to the dissociation of ligands under operation as supported by the different binding energies obtained in our calculation. We sought to quantitatively investigate the trap density and energy evolved in the QDs devices by TAS^11,30,31^. In the TAS measurements, the dynamic response of traps with respect to the modulated AC electric field would attribute to additional capacitance on the device dielectric response. Such traps induced capacitance strongly depends on the DOS and energy of trap charges across the QDs bandgap. Briefly, trapped charges near the band edge have higher probability to jump to the conduction/valance band, which could follow the AC electric field at higher frequency and contribute to the capacitance increment. However, the contribution from deeper traps can be observed at lower frequency. The correlation between the DOS of trap charges can the frequency dependent capacitance that can be expressed as refs. ^30,31^:

1 $$N_{\rm{t}}\left( {E_{\rm{w}}} \right) = - \frac{{V_{\rm{bi}}^2}}{{w\left[ {qV_{\rm{bi}} - \left( {E_{\rm{f}} - E_{\rm{w}}} \right)} \right]}}\frac{{\partial C}}{{\partial w}}\frac{w}{{kT}},$$

where *N*_t_ is the DOS of trap charges, *V*_bi_ is the built-in potential, *w* is the depletion width of the QDs devices, *E*_f_ is the Femi level, and *E*_w_ is the energy level of the trap state relative to the valence band. The calculated trap charges DOS of QDs with different passivation strategies is illustrated in Fig. 8b. For the Zn(OA)_2_-QDs device, it has ultralow DOS of traps through the whole bandgap. Strikingly, even after aging, the DOS of traps remains around 1.0 × 10^16^ cm^−3^ eV^−1^. This is consistent with the simulation results that the dual surface passivation ability of Zn(OA)_2_ facilitates strong binding energy as compared with other ligands. On the other hand, both OA-QDs and ZnCl_2_-QDs devices have higher trap DOS in fresh samples and increased after aging. Particularly, the OA-QDs devices has a noticeable increase of deep traps at 0.40–0.55 eV, and the ZnCl_2_-QDs devices has shallower traps emerged around 0.35 eV and increased after aging. The increase of trap DOS in OA-QDs and ZnCl_2_-QDs devices at different energies is related to the passivation effect on different surface terminations, which is also demonstrated in the simulation results.

## Discussion

In conclusion, we investigated the ligand effects on the device performance in perspective of surface trap states. Through simulation, we found that both ionic sites of shell surface need to be passivated to reduce the trap states. Based on the requirement of IJP process, we proposed a dual ionic passivation method by liquid phase ligand exchange using Zn(OA)_2_ organic salt. By successfully suppressing the mid-gap trap states, our Zn(OA)_2_-QDs based IJP QLEDs show high EQE of 16.6% and long lifetime of 1,721,000 h. The IJP QLED performances we reported here start to meet the basic requirement of industry application and the methods could be extended to other CQD materials and devices as well.

## Methods

### Material synthesis

The quantum dots used in this work were prepared according to the methods previously reported in the related literatures with appropriate modifications^2^. For a typical synthesis of CdSe/Cd1-xZnxSe/ZnSe QDs, 0.4 mmol of CdO, 6 mmol of zinc acetate, and 7 ml of OA were placed in a 50 ml flask and heated to 170 °C in flowing high-purity argon for 30 min. Then 15 ml of 1-octadecene (ODE) was added to the flask and the temperature was elevated to 300 °C. A stock solution containing 0.9 mmol of Se dissolved in 2 ml of trioctylphosphine (TOP) was quickly injected into the flask. The reaction temperature was kept for 10 min. Then 0.1 mmol Se dissolved in 1 ml TOP was injected into the flask at the elevated temperature of 300 °C and reacted for 10 min. Finally, the reaction mixture was cooled to room temperature. For a typical ligand exchanged process, the as-synthesized QDs (1 g) were dissolved in a octadecane solution of oleyl amine (25 ml, 0.5 M) and stirred at 100 °C for 4 h to remove the originally coated OA ligands. Then ethanol was added to a total volume of 50 ml, causing precipitation of the QDs. After separation of the QDs by centrifugation (7300 rpm for 5 min), the QDs were dissolved in 25 ml of octadecane and precipitated a second time with ethanol again. The whole washing process would repeat for five times. After washing, the P-QDs were stored in octane, denoted as P-QDs, and were ready for the further treatment. For the next step of the solution ligand exchange procedure, the specific surface ligand, such as OA, ZnCl_2_, and Zn(OA)_2_, was also dissolved in octadecane (25 ml, 0.5 M for OA/0.05 M for ZnCl_2_/0.25 M for Zn(OA)_2_) at 200 °C and then added into the P-QDs solution (25 ml, 1 g). After stirred for 4 h at 200 °C and then washed with ethanol for five times, the aimed ligand coated QDs were achieved and stored in octane. The solvents of the QDs ink follow the protocols from reference^32^. To formulate QD ink, red QDs powders were dispersed in the mixed solvents of Cyclohexylbenzene and decane with a volume ratio of 9:1, and a concentration of 25 mg ml^−1^.

ZnO nanoparticles were synthesized by a solution-precipitation process reported in the literature^3^. For a typical synthesis, a solution of zinc acetate in dimethyl sulfoxide (0.5 M) and 30 ml of a solution of tetramethylammonium hydroxide in ethanol (0.55 M) were mixed and stirred for 1 h in ambient conditions, then washed and dispersed in ethanol. To formulate ZnO ink, the ZnO nanoparticles were dispersed in the mixed solvents of n-octanol and n-butanol with a volume ratio of 7:3, and a concentration of 30 mg ml^−1^.

### Device fabrication

QLED devices were fabricated by consecutively IJP each layer on glass substrates. Glass substrates were pre-coated with an ITO anode (sheet resistance is about 50 Ω ϒ^−1^). Prior to use, all substrates were cleaned by ultrasonic treatment in tergitol/DI-water solution, DI-water, and isopropanol, respectively, followed by treating under UV-ozone for 15 min. A 25-nm-thick Nissan HIL layer was first IJP onto the cleaned substrates, then substrate was transfer to a vacuum chamber dry (VCD) system with a 3 Pa pressure for 10 min and annealed at 190 °C in air to remove residual water. A 40-nm-thick TFB (American Dye Source) layer was then deposited in a N_2_ glovebox, followed by VCD process with a 1000 Pa pressure for 10 min and a thermal anneal at 200 °C for 30 min. After that, a QD layer and a ZnO nanoparticle layer were sequentially IJP in the glovebox, respectively, then VCD with 3 Pa for 10 min and annealed in the same environment at 120 °C to remove the residual solvents. Thicknesses of the QD layer and ZnO layer were varied by changing the ink drops. The optimal thicknesses for QD layers and ZnO layers were 20 and 40 nm, respectively. Supplementary Fig. 7 shows the comparison of surface roughness of each QDs, and Supplementary Fig. 8 shows the example measurement of QD and ZnO layers thicknesses. After the IJP of the solution-processed layers, all samples were transferred to a vacuum deposition chamber with chamber pressure <10^−6^ torr (*P* < 10^−6^ torr) for Al cathode (100-nm thick) deposition, followed by the final encapsulation with a UV-curable epoxy and cover glasses in the N_2_ glovebox.

### Characterizations

Photoluminescence spectra of QDs were obtained in octane solutions with an Edinburgh FS5 steady state fluorescence spectrometer by excitation at the absorption maxima. The absolute quantum yield of QDs solutions was measured using the built-in integrating sphere of Edinburgh spectrometer. TEM images were taken using a JEOL-2100F TEM (Shanghai Your Equipment Technology Co. Ltd) with 200 keV electron beam energy, and XRD patterns were taken using a Buker D8 Advance Diffractometer (Shanghai Your Equipment Technology Co. Ltd). XPS data were collected with a PHI 5000 VersaProbe X-ray photoelectron spectrometer using monochromatic X-rays from an Al anode. High-resolution data were collected from the region around Zn 2*p* and Se 3*d*. Atomic force microscopy (AFM) topographies of ITO/PEDOT:PSS/TFB/QDs films were carried out on a Bruker Dimension ICON AFM (Bruker Corp., Billerica, Massachusetts, United States). FTIR was conducted by using the Thermo Scientific Nicolet™ 6700 FT-IR spectrometer. NMR samples were dissolved in CDCl_3_ and were characterized by Bruker Avance III HD 400. Electroluminescence spectra were obtained using an Ocean Optics USB 2000+ spectrometer with the devices driven at a constant current with a Keithley 2400 source meter. The J–L–V characteristics of the devices were taken under ambient conditions with a Keithley 2400 source meter measuring the sweeping voltages and currents and a Keithley 6485 Picoammeter together with a calibrated silicon detector (Edmund) measuring light intensities. The EQE (as photons per electron) was calculated by converting the photo current to emitted photons and the device current to electrons simultaneously^33,34^. Luminance was calibrated using a photometer (Spectra Scan PR655) with the assumption of Lambertian emission pattern of all devices. The lifetime test was conducted under ambient conditions using a commercialized lifetime test system (Guangzhou New Vison Opto-electronic Technology Co. Ltd). The TAS of the devices were conducted inside a temperature regulated cryostat in vacuum. The sample temperature was measured with a PT100 sensor directly attached on the sample substrate. An Agilent 4284A LCR meter was used to measure the device capacitance. In the measurement, an AC modulation voltage of 50 mV was applied on the QDs devices to excite the dynamic response of traps at different frequencies.

## Supplementary information

Supplementary Information

## Data Availability

All data are available in the manuscript or the supplementary materials.

## References

[1] Yang Y (2015). High-efficiency light-emitting devices based on quantum dots with tailored nanostructures. Nat. Photonics.

[2] Cao W (2018). Highly stable QLEDs with improved hole injection via quantum dot structure tailoring. Nat. Commun..

[3] Qian L, Zheng Y, Xue J, Holloway PH (2011). Stable and efficient quantum-dot light-emitting diodes based on solution-processed multilayer structures. Nat. Photonics.

[4] Shirasaki Y, Supran GJ, Bawendi MG, Bulović V (2013). Emergence of colloidal quantum-dot light-emitting technologies. Nat. Photonics.

[5] Nurmikko A (2015). What future for quantum dot-based light emitters?. Nat. Nanotechnol..

[6] Mcdonald SA (2005). Solution-processed PbS quantum dot infrared photodetectors and photovoltaics. Nat. Mater..

[7] Clifford JP (2009). Fast, sensitive and spectrally tuneable colloidal-quantum-dot photodetectors. Nat. Nanotechnol..

[8] Wang R (2016). Colloidal quantum dot ligand engineering for high performance solar cells. Energy Environ. Sci..

[9] Tang J (2011). Colloidal-quantum-dot photovoltaics using atomic-ligand passivation. Nat. Mater..

[10] Ning Z (2012). All-inorganic colloidal quantum dot photovoltaics employing solution-phase halide passivation. Adv. Mater..

[11] Ip AH (2012). Hybrid passivated colloidal quantum dot solids. Nat. Nanotechnol..

[12] Liu M (2017). Hybrid organic-inorganic inks flatten the energy landscape in colloidal quantum dot solids. Nat. Mater..

[13] Talapin DV, Steckel J (2013). Quantum dot light-emitting devices. MRS Bull..

[14] Dai X, Deng Y, Peng X, Jin Y (2017). Quantum-dot light-emitting diodes for large-area displays: towards the dawn of commercialization. Adv. Mater..

[15] Xiang C, Cao W, Yang Y, Qian L, Yan X (2018). The dawn of qled for the FPD industry. Inf. Disp..

[16] Jiang C (2016). Coffee-ring-free quantum dot thin film using inkjet printing from a mixed-solvent system on modified ZnO transport layer for light-emitting devices. ACS Appl. Mater. Interfaces.

[17] Singh M, Haverinen HM, Dhagat P, Jabbour GE (2010). Inkjet printing-process and its applications. Adv. Mater..

[18] Chen, P. et al. 65-Inch inkjet printed organic light-emitting display panel with high degree of pixel uniformity. *SID Int’l Symp Dig Tech Pap*. **45**, 396–398 (2014).

[19] Li X (2018). Bright colloidal quantum dot light-emitting diodes enabled by efficient chlorination. Nat. Photonics.

[20] Boles MA, Ling D, Hyeon T, Talapin DV (2016). Erratum: the surface science of nanocrystals. Nat. Mater..

[21] Anderson NC, Hendricks MP, Choi JJ, Owen JS (2013). Ligand exchange and the stoichiometry of metal chalcogenide nanocrystals: spectroscopic observation of facile metal-carboxylate displacement and binding. J. Am. Chem. Soc..

[22] Brown PR (2014). Energy level modification in lead sulfide quantum dot thin films through ligand exchange. ACS Nano.

[23] Chuang CHM, Brown PR, Bulović V, Bawendi MG (2014). Improved performance and stability in quantum dot solar cells through band alignment engineering. Nat. Mater..

[24] Shen H (2015). High-efficiency, low turn-on voltage blue-violet quantum-dot-based light-emitting diodes. Nano Lett..

[25] Kang BH (2016). Efficient exciton generation in atomic passivated CdSe/ZnS quantum dots light-emitting devices. Sci. Rep..

[26] Ning Z (2014). Air-stable n-type colloidal quantum dot solids. Nat. Mater..

[27] Owen J (2015). The coordination chemistry of nanocrystal surfaces. Science.

[28] Vincent Crist, B. Handbooks of monochromatic XPS spectra volume 1—the elements and native oxides. in *Handbook of The Elements and Native Oxide*, Vol. 1, 1–87 (XPS International LLC, 1999).

[29] Shen H (2019). Visible quantum dot light-emitting diodes with simultaneous high brightness and efficiency. Nat. Photonics.

[30] Walter T, Herberholz R, Müller C, Schock HW (1996). Determination of defect distributions from admittance measurements and application to Cu(In,Ga)Se2 based heterojunctions. J. Appl. Phys..

[31] Herberholz R, Igalson M, Schock HW (1998). Distinction between bulk and interface states in CulnSe2/CdS/ZnO by space charge spectroscopy. J. Appl. Phys..

[32] Liu Y (2017). Efficient all-solution processed quantum dot light emitting diodes based on inkjet printing technique. ACS Appl. Mater. Interfaces.

[33] Forrest SR, Bradley DDC, Thompson ME (2003). Measuring the efficiency of organic light-emitting devices. Adv. Mater..

[34] Mashford BS (2013). High-efficiency quantum-dot light-emitting devices with enhanced charge injection. Nat. Photonics.

