# Supplementary material for: High efficiency and stability of ink-jet printed quantum dot light emitting diodes
Source: Nat Commun. 2020 Apr 2;11:1646. doi: 10.1038/s41467-020-15481-9 (PMC7118149; doi:10.1038/s41467-020-15481-9)
Supplement: Supplementary file 1 — Supplementary Information [file 41467_2020_15481_MOESM1_ESM.pdf]

Supplementary Information

High efficiency and Stability of Ink-jet Printed Quantum Dot Light Emitting Diodes

Xiang et al.

Supplementary Figures:

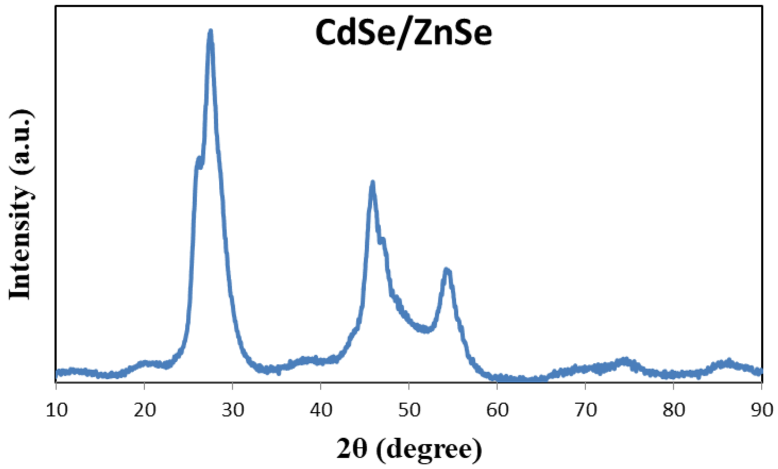

Supplementary Figure 1, the XRD measurement of QDs used in this study. The characteristic peaks indicate the wurtzite crystal structure of our QDs. The XRD result matches the XRD peaks of wurtzite ZnSe from the two references[1,2].

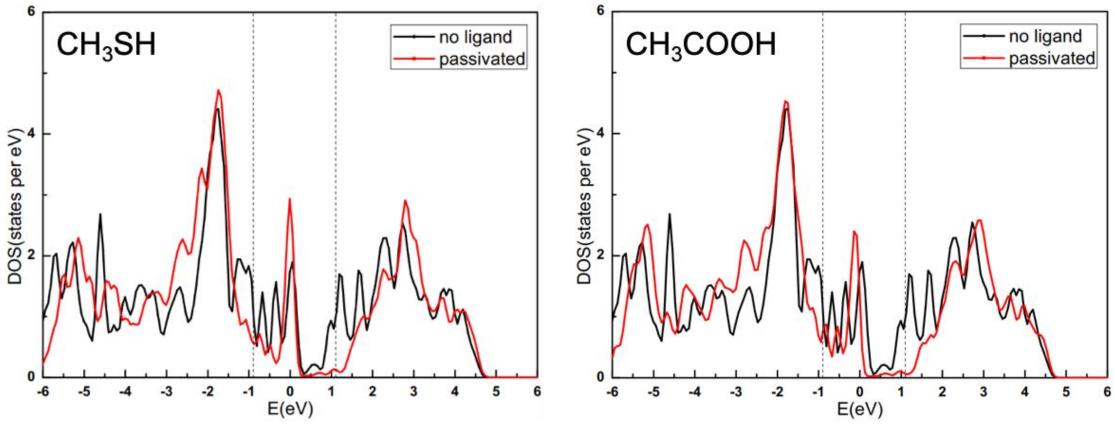

Supplementary Figure 2, the simulated DOS before and after passivation using CH_3SH and CH_3COOH.

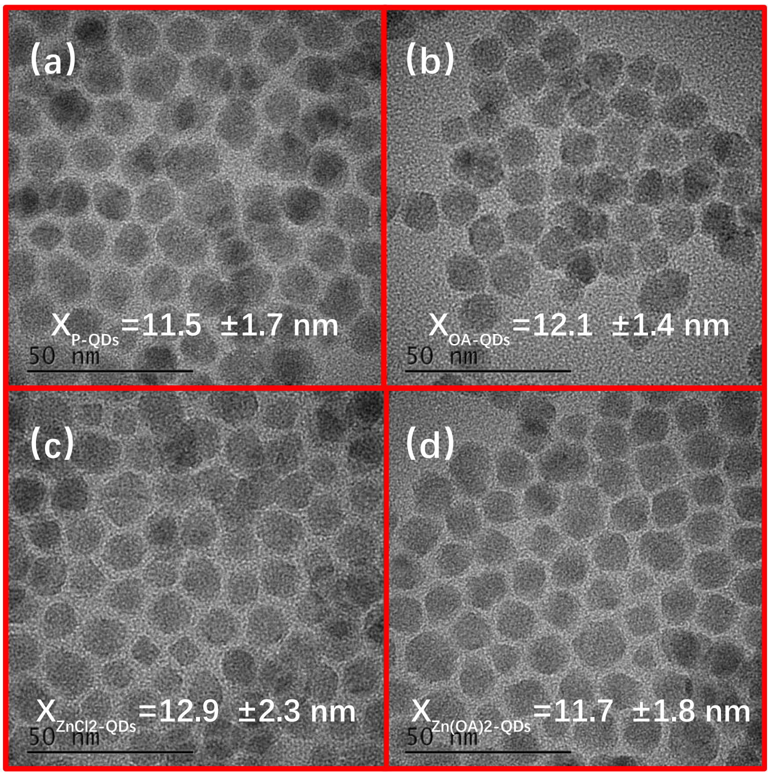

Supplementary Figure 3, TEM images with scale bar of 50nm. (a) P-QDs, (b) OA-QDs, (c) $ZnCl_2$-QDs, and (d) $Zn(OA)_2$-QDs. Every image labeled with the average particle size of the corresponding ligand-exchanged QDs.

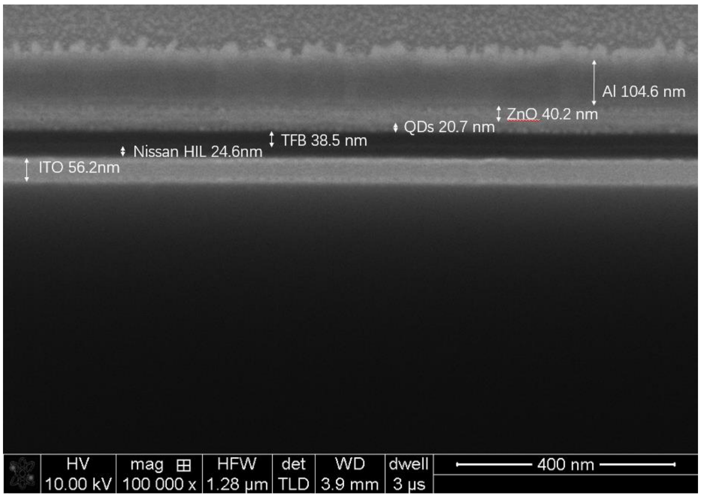

Supplementary Figure 4, the SEM of a typical $Zn(OA)_2$-QDs device.

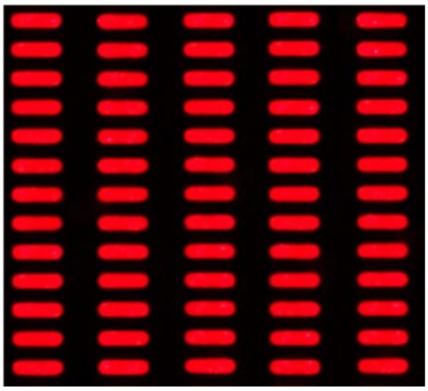

Supplementary Figure 5, the pixel array image of a typical Zn(OA)$_2$-QDs device.

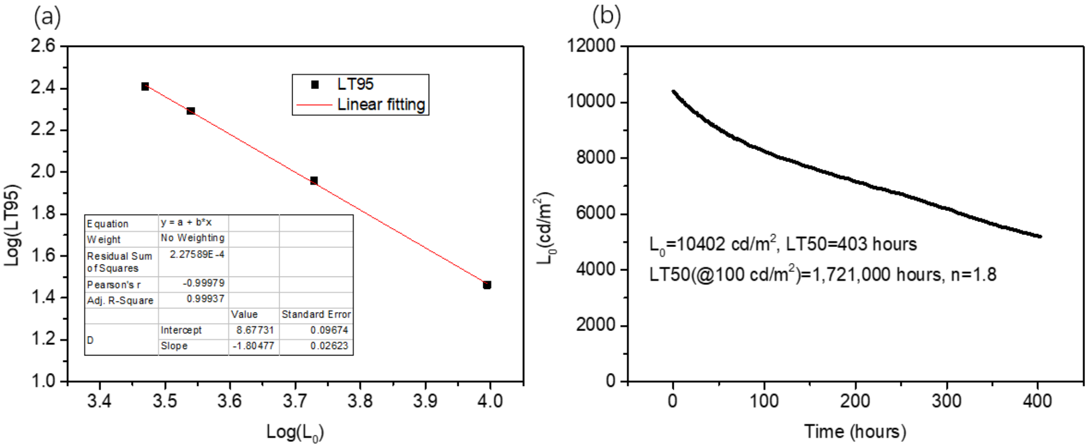

Supplementary Figure 6, accelerating factor (n) for the lifetime and LT$_{50}$ lifetime. (a), Extrapolation of accelerating factor (n) for the lifetime estimation by fitting the Log(LT_{95})-Log(L_0) data points. Lifetime test was conducted under accelerated conditions to shorten the testing period, as commonly used in OLEDs. The initial luminance (L) and the measured time (t) for certain luminance degradation follows the empirical formula: $L^n \bullet t$ = constant. Here, n is defined as the accelerating factor, which can be obtained by fitting the values at multiple initial luminance. The lifetime at low luminance can therefore be extrapolated from the lifetime of the same device operated at high luminance following the equation. In our case, the accelerating factor is 1.80.

; (b), LT_{50} lifetime of a Zn(OA)$_2$-QDs device operated at constant current density of 100 mA cm^{-2}.

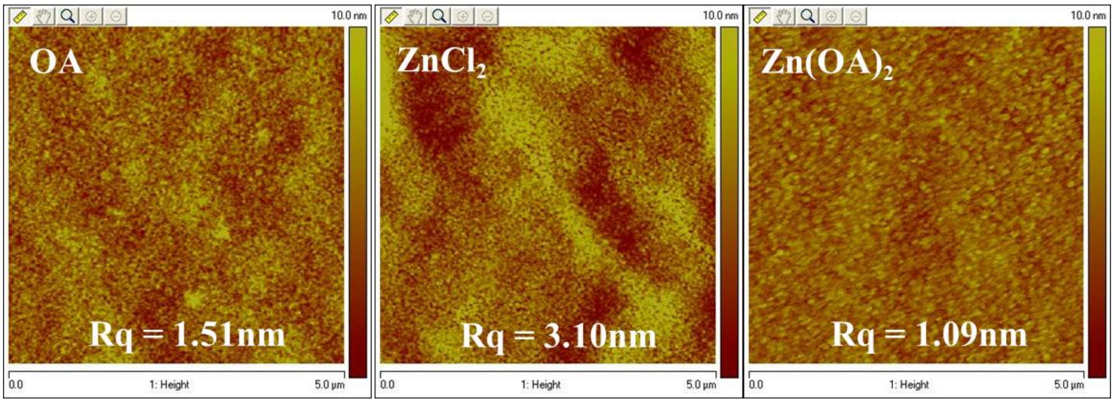

Supplementary Figure 7, the AFM images of ink-jet printed QD films. QDs were ink-jet printed on top of ITO/PEDOT:PSS/TFB, the device structure that we used in this work, with different types of ligands. It is seen that among the QDs films with three different types of ligands, QDs film with $Zn(OA)_2$ ligand has shown the best film surface roughness. Rq has been defined as root mean square roughness

(a)

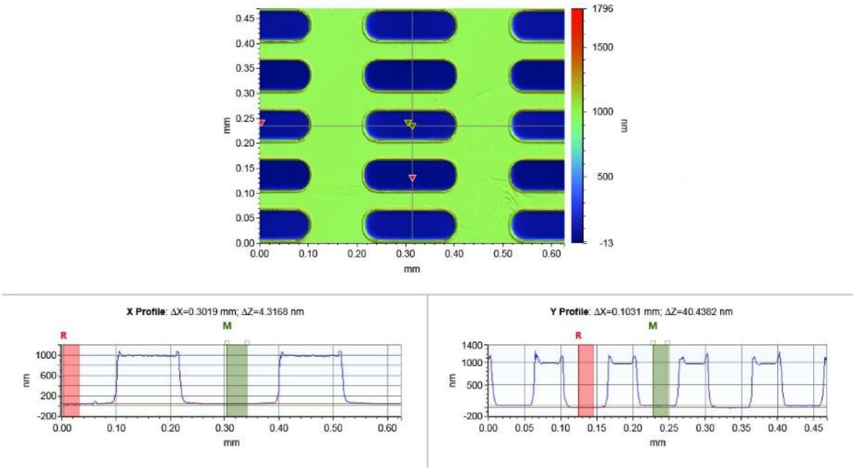

(b)

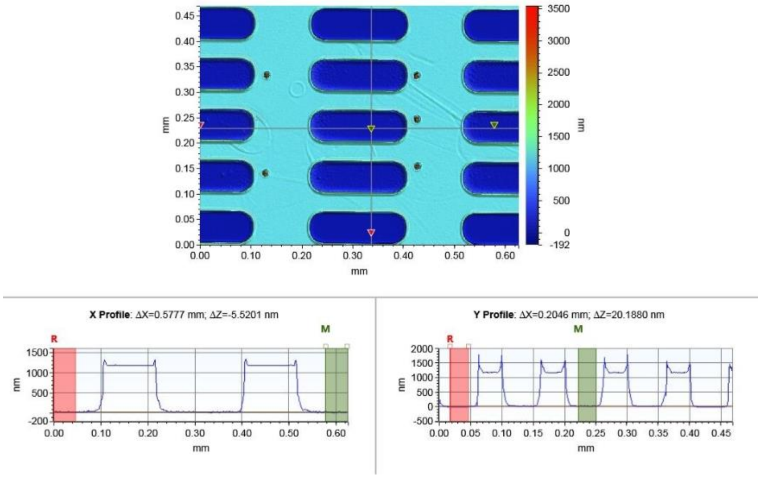

Supplementary Figure 8, the measurements of ZnO and QD layer thicknesses. The measurements of ZnO (a) and QD (b) layer thicknesses of a typical $Zn(OA)_2$-QDs device by white light interference. The statistic bias is 40.4±1.3 nm and 20.2±0.8 nm for ZnO and QD respectively.

Supplementary References:

1. Senthilkumar, K., Kalaivani, T., Kanagesan, S., Balasubramanian, V. & Balakrishnan, J. Wurtzite ZnSe quantum dots: Synthesis, characterization and PL properties. *J. Mater. Sci. Mater. Electron.* **1**, 59–69 (2013).
2. Chen, H. S. *et al.* Colloidal ZnSe, ZnSe/ZnS, and ZnSe/ZnSeS quantum dots synthesized from ZnO. *J. Phys. Chem. B* **108**, 17119–17123 (2004).
